# Vaginal prevention of *Candida albicans*: synergistic effect of lactobacilli and mannan oligosaccharides (MOS)

**DOI:** 10.1007/s00253-023-12909-2

**Published:** 2024-01-09

**Authors:** Margarida Faustino, Joana Odila Pereira, Ana Margarida Pereira, Ana Sofia Oliveira, Carlos M. H. Ferreira, Carla F. Pereira, Joana Durão, Manuela E. Pintado, Ana P. Carvalho

**Affiliations:** 1 Universidade Católica Portuguesa, CBQF- Centro de Biotecnologia E Química Fina - Laboratório Associado, Escola Superior de Biotecnologia, Rua Diogo Botelho 1327, 4169-005 Porto, Portugal; 2 Amyris Bio Products Portugal Unipessoal Lda, Porto, Portugal

**Keywords:** Mannan oligosaccharides, *Lactobacillus crispatus*, HeLa cells, Competition assays, Prophylaxis assays, Vaginal therapeutics

## Abstract

**Abstract:**

Vulvovaginal candidiasis (VVC) affects approximately 30–50% of women at least once during their lifetime, causing uncomfortable symptoms and limitations in their daily quality of life. Antifungal therapy is not very effective, does not prevent recurrencies and usually causes side effects. Therefore, alternative therapies are urgently needed. The goal of this work was to investigate the potential benefits of using mannan oligosaccharides (MOS) extracts together with a *Lactobacillus* sp. pool, composed by the most significant species present in the vaginal environment, to prevent infections by *Candida albicans.* Microbial growth of isolated strains of the main vaginal lactobacilli and *Candida* strains was assessed in the presence of MOS, to screen their impact upon growth. A pool of the lactobacilli was then tested against *C. albicans* in competition and prophylaxis studies; bacterial and yeast cell numbers were quantified in specific time points, and the above-mentioned studies were assessed in simulated vaginal fluid (SVF). Finally, adhesion to vaginal epithelial cells (HeLa) was also evaluated, once again resorting to simultaneous exposure (competition) or prophylaxis assays, aiming to measure the effect of MOS presence in pathogen adherence. Results demonstrated that MOS extracts have potential to prevent vaginal candidiasis in synergy with vaginal lactobacilli, with improved results than those obtained when using lactobacilli alone.

**Key points:**

- *Potential benefits of MOS extracts with vaginal lactobacilli to prevent C. albicans infections.*
- *MOS impacts on growth of vaginal lactobacilli pool and C. albicans in SVF.*
- *MOS extracts in synergy with L. crispatus inhibit C. albicans adhesion in HeLa cells.*

**Graphical Abstract:**

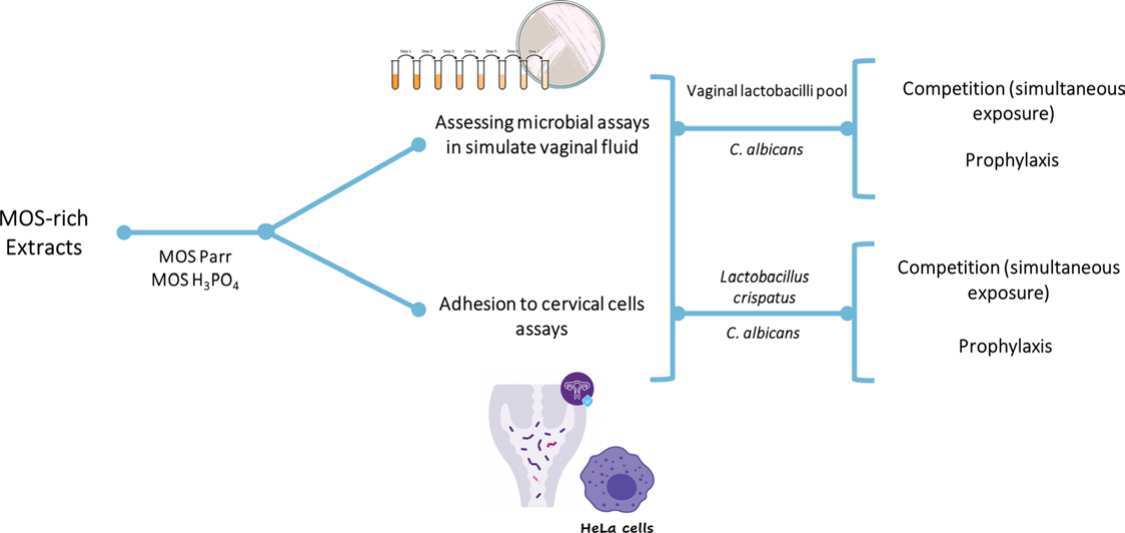

**Supplementary Information:**

The online version contains supplementary material available at 10.1007/s00253-023-12909-2.

## Introduction

Worldwide, a percentage of about 30 to 50% of women experience vulvovaginal candidiasis (VVC) at least once during their life span (Corsello et al. 2003; Foxman et al. 2013); in many situations their appearance is even recurrent (Sobel 2014). The aforementioned infection elicits a multitude of symptoms, including a thick and white vaginal discharge, dyspareunia, vulvar pruritus, erythema and swelling (Falagas et al. 2006; Al-Ghazzewi and Tester 2016). An increased amount of studies highlights the significant impact of fungal infections on women’ life quality, emphasizing the importance to optimize the management and/or treatment of patient management and treatment for those diagnosed with VVC (Aballéa et al. 2013). Therefore, the study of alternative strategies to either replace or combine with standard therapies aims to achieve more efficient prevention and/or treatment of this highly relevant vaginal infection.

VVC is mainly caused by *Candida* species, mainly *Candida albicans*, *Candida glabrata*, or *Candida krusei* (Jacob et al. 2018). The currently available antifungal treatments and antifungal agents often lead to deficient healing and recurrent infections, due to acquired antimycotic resistance (van de Wijgert and Verwijs 2020; Zangl et al. 2020). Alternative strategies may include substances that aim the re-establishment of a healthy vaginal environment such acidifying agents, probiotics, and prebiotics, while triggering an improved local immunity response.

In healthy women, the vaginal microbiota is typically composed of a variety of anaerobic and aerobic microorganisms. Among these, lactobacilli are the most common and often the predominant microorganisms, playing a crucial role as a protective barrier against infections. The ability of lactobacilli to adhere to and compete for adhesion sites on the vaginal epithelium, in addition to their capacity to produce antimicrobial molecules such as hydrogen peroxide, lactic acid, and bacteriocin-like substances (BLS), are pivotal in the prevention the pathogens’ new colonization (Borges et al. 2014). Six types of vaginal microbiota, named community state types (CSTs), have been delineated: CST I is dominated by *Lactobacillus crispatus*, CST II by *Lactobacillus gasseri*, CST III by *Lactobacillus iners*, CST IV A and CST IV-B mainly by anaerobic bacteria, and CST V by *Lactobacillus jensenii* (Lacroix et al. 2020).

The pro- and prebiotic approaches have been extensively studied in the treatment of gastrointestinal infectious diseases, inflammatory bowel disease (such as ulcerative colitis) (Lin et al. 2008), Crohn’s disease (De Vrese and Schrezenmeir 2008), and in prevention of colon cancer (Liong 2008). Both topical and oral probiotics have been commonly indicated for vaginal health (Hilton et al. 1992; Coste et al. 2012), supporting the premise that vaginal microbiota can be restored after oral intake of lactobacilli. Indeed, various clinical studies have demonstrated the efficacy of probiotics in the treatment and prevention of vaginal infections such as bacterial vaginosis (Homayouni et al. 2014), vulvovaginal candidiasis (Falagas et al. 2006), and urinary tract infections (Borchert et al. 2008) when administered topically. The utilization of vaginal probiotics is based on the importance of maintaining a healthy vaginal microbiota and the necessity to restore the microbial ecosystem following disturbances (Mastromarino et al. 2013).

A potential alternative to the probiotic treatments is the use of prebiotic compounds, owing to their possible local microflora stimulus effect. To our utmost understanding, there are only a limited number of reports regarding the utilization of prebiotic carbohydrates in the treatment of vaginal infections. Tester and Al-Ghazzewi (2018) reported the use of oligosaccharides or polysaccharides (derived from mannose, fructose, glucose, galactose, and uronic acids) locally delivered in the form of pessaries in order to stimulate the selective growth of healthy vaginal bacteria. The introduction of these carbohydrates into the vagina aims to provide prebiotics that are selectively utilized by lactic acid bacteria, inhibiting the pathogens’ growth (Tester and Al-Ghazzewi 2018). These pathogens include *Gardnerella* spp., *Prevotella* spp., *Mobilincus* spp., *Megaspahera* spp., *Sneathea* spp., and various anaerobic species associated with bacterial vaginosis (BV) (Mashatan et al. 2023). Importantly, prebiotics play a crucial role in fostering the growth of beneficial lactobacilli, thereby contributing significantly to preventing the proliferation of pathogens such as *Atopobium vaginae*, *Megasphaera* spp., and *Sneathea* spp. This becomes particularly pertinent in individuals at a heightened risk of sexually transmitted infections (Zhang et al. 2020). Furthermore, prebiotics, specifically in the form of oligosaccharides or polysaccharides, have been observed to actively assist in averting the overgrowth and colonization of *Candida* species, including *C. albicans*, *C. glabrata*, *C. parapsilosis*, *C. tropicalis*, and *C. krusei*. The strategic combination of prebiotics and probiotics emerges as a suggested and effective approach for reducing and managing *Candida* fungus. This holistic intervention holds promise in not only preventing the overgrowth of vaginal pathogens but also in fostering a balanced and resilient microbiome (Ohshima et al. 2016).

Sutherland et al. (2009) have also shown the preventive potential of topical application of prebiotics on vaginal infections, namely bacterial vaginosis and candidiasis. According to Rousseau et al. (2005), fructo-oligosaccharides and gluco-oligosaccharides have been reported to have a positive impact on the growth of vaginal lactobacilli based on in vitro models. In a clinical study conducted by Coste et al. (2012), the use of gels containing gluco-oligosaccharides resulted in the restoration of normal vaginal flora in treated patients. These findings suggest that fructo-oligosaccharides and gluco-oligosaccharides may have beneficial effects on vaginal lactobacilli growth and the recovery of a healthy vaginal microbiota. In a related research direction, Rousseau et al. (2005) and Bou-Antoun (2011) investigated the in vitro impact of fructo- and galacto-oligosaccharides on vaginal lactobacilli, such as *Lactobacillus acidophilus*, *Lactobacillus casei*, and *Lactobacillus fermentum*. These oligosaccharides were utilized to promote the growth of beneficial vaginal microflora and regulate the proliferation of pathogens.

In this work, the feasibility of using yeast mannan oligosaccharides (MOS) as supplements for the management of candidiasis was evaluated by assessing its effect on *Lactobacillus* sp. and *C. albicans*. Competition and prophylaxis assays were performed in simulated vaginal fluid (SVF). Additionally, MOS effect on the adhesion to HeLa vaginal cells of both *Lactobacillus* sp. and *C. albicans* was studied also in competition and prophylaxis simulation assay.

## Materials and methods

### MOS extracts, commercial benchmark, culture media, and microorganisms

The MOS extracts utilized in this study were procured from genetically modified spent yeast (*Saccharomyces cerevisiae*) that were obtained from Amyris, Inc (Emeryville, California, USA). The extraction protocol involved a hydrothermal treatment at a temperature of 110 °C during 3 h (MOS Parr) and an acidic treatment that included the use of phosphoric acid at a temperature of 55 °C for 24 h (MOS H_3_PO_4_). D-mannose was acquired from Sigma-Aldrich. All microorganisms (listed in Supplementary Table S1) were acquired from Deutsche Sammlung von Mikroorganismen und Zellkulturen (DSMZ, Braunschweig, Germany). Different culture media used were according to the assay requirements: (i) Broth and agar De Man, Rogosa and Sharpe (MRS, Biokar Diagnostics, Allone, France) for *Lactobacillus* sp.; (ii) Yeast and Mould broth (YM, Sigma-Aldrich, St. Louis, MO, USA) for growth of *Candida* sp.; (iii) Simulated vaginal fluid (SVF) pH 4.2 (reagents used are listed in Supplementary Table S2) according to the recipe described in Owen and Katz (1999); and (iv) Chromogenic *Candida* agar (CHROMagar Candida, Frilabo, Maia, Portugal).

### Inoculum preparation

*L. crispatus*, *L. gasseri*, and *L. jensenii* were grown at 37 °C in anaerobic conditions in modified MRS, with 5 g/L of glucose and 0.05% l-cysteine hydrochloride (Sigma-Aldrich, St. Louis, MO, USA). The amount of glucose was reduced from the original recipe to mimic the sugar content of SVF, whereas cysteine was added due to *L. crispatus* nutritional requirements, according to DSMZ recommendations. *C. albicans*, *C. glabrata*, and *C. tropicalis* were grown at 30 °C in aerobic conditions in YM broth.

### MOS antifungal activity against *Candida* sp.

The study of the potential antifungal activity of the different samples (MOS H_3_PO_4_, MOS Parr, and D-mannose) was performed in modified Muller Hinton (MH) (CLSI 2012). Natamycin (Sigma-Aldrich, St. Louis, USA) was used as positive control, while MH medium was used as negative control. All samples and natamycin were diluted at 2% (w/v) in MH medium and sterilized using a sterile 0.22 µm filter (Millipore, Billerica, MA, USA).

The above-mentioned *Candida* strains were grown as a monoculture before the assay in YM broth at 30 °C for 24 h under aerobic conditions, and subsequently cultivated in solid media. Subsequently, a single colony was selected, resuspended in 10 mL of MH broth, and then cultivated under aerobic conditions at 37 °C during 24 h. The inoculum was adjusted to an optical density (OD) at 625 nm of 0.1–0.08 (equivalent to a cell density of 1 × 10^8^ cells/mL) and diluted by a factor of 10 to obtain the working inoculum. To assess bacterial growth inhibition, 980 µL of each sample were transferred to a sterile microtube and inoculated with 20 µL of the working inoculum. Bacteria were then transferred to a 96-well microplate (Nunc, Darmstadt, Germany) and incubated for 48 h. Bacterial growth was monitored at 625 nm and 37 °C (hourly intervals) for a period of 48 h, using a microplate reader (Epoch, Vermont, USA). Sample blanks were used to compensate for any interference in sample color and optical density (OD).

### Assessing microbial growth in simulated vaginal fluid: competition and prophylaxis assays

#### *Lactobacillus* sp. pool preparation

Despite the referred prevalence of *L. crispatus*, *L. gasseri*, and/or *L. jensenii* in vaginal environmental, this is not a consensual issue, since vaginal microbiome may change between individual women but also depend on age and ethnicity, among others (Zangl et al. 2020). Therefore, to simulate the predominant environment in the vaginal flora of the *Lactobacillus* sp. population, a mix of the three strains of vaginal lactobacilli (*L. crispatus*, *L. gasseri*, and *L. jensenii*) in a ratio of 1:1:1 was used, with a final OD of 0.1 at 625 nm.

#### Simulated vaginal fluid

SVF was prepared according to the procedure from Owen and Katz (1999) with a final pH of 4.2. This pH value was selected to replicate the vaginal pH in physiological conditions. The solution was sterilized at 121 °C for 20 min before adding 0.05% l-cysteine hydrochloride, previously sterilized through filtration using a 0.22 µm filter (Millipore, Billerica, MA, USA).

#### Microbial growth curves with isolated strains

The effect of the different MOS extracts on microbial growth was determined by screening their impact upon the growth curves of *Lactobacillus* sp. (*L. crispatus*, *L. gasseri*, and *L. jensenii*) and vaginal pathogens (*C. glabrata, C. albicans* and* C. tropicalis*) in modified SVF. This preliminary assay allowed to better design the subsequent prophylaxis and competition assays, in which both *Lactobacillus* and *Candida* are used simultaneously.

The *Lactobacillus* sp. strains and *Candida* sp. strains were grown as described in section “inoculum preparation.” The inoculums were centrifuged after 24 h of growth (4700 × g, 5 min, 4 °C), washed twice with phosphate-buffered saline, pH 7.4 (PBS) and resuspended in the same initial volume with SVF. To obtain a standard concentration in each condition, inoculums were diluted to an OD of 0.1 at 625 nm, to which 2% (v/v) of each extract, previously prepared by dissolving MOS in SVF at 2% (w/v) and sterilizing with a 0.22-µm pore size filter, was added. The assay was performed in a 96-well microplate (Nunc, Darmstadt, Germany) to which 200 µL of each sample condition were transferred. Additionally, 50 µL of paraffin were added to ensure the anaerobic environment. The microplates were incubated for 48 h at 37 °C, and absorbance was measured at 625 nm, every hour, with a multidetector plate reader (Epoch, Vermont, USA). SVF medium was used as negative control. The D-mannose was used as a commercial benchmark against MOS extracts (since these are mainly composed by mannose oligomers), also at 2% (w/v) in SVF. The experiment was performed in triplicates.

#### Prophylaxis assay (sequential exposure) with SVF

Each of the three *Lactobacillus* inoculums was adjusted to an OD (625 nm) of 0.1 and then mixed in a proportion of 1:1:1. This pool was allowed to grow in SVF for 16 h in anaerobiosis (selected time based on the results from Supplementary Data Figure S1) with the different extracts at a concentration of 2% (w/v). At the end of the 16 h, the *C. albicans* inoculum (OD of 0.1, at 625 nm) was added at 2% (v/v). Sampling points were carried out at 0 h and 16 h pre-infection, and then at 18 h, 20 h, 24 h, and 40 h after infection with *C. albicans*. Sequential tenfold dilutions were carried out in sterile peptone water (Sigma-Aldrich, St. Louis, USA) and plated (in quadruplicate), using the drop technique (Miles et al. 1938). MRS agar with 98 mg/L of fluconazole (to inhibit *Candida* sp. growth) was used for *Lactobacillus* sp. counting, while CHROMagar was used for *C. albicans* counting, incubated at 37 °C under anaerobic conditions and 30 °C under aerobic conditions, respectively.

#### Competition assay (simultaneous exposure) with SVF

Except for adding the *Lactobacillus* sp. pool and *C. albicans* to SVF at the same time (both at 2% (v/v)) followed by incubation at 37 °C in anaerobic conditions, this assay was performed as described for the prophylaxis assay. Sampling points were carried out, at 0 h, 2 h, 4 h, 8 h, 24 h, and 48 h. Microorganism counting methodology was the performed as described in section “prophylaxis assay (sequential exposure) with SVF.”

### Adhesion to cervical cells

#### Cell line growth conditions

Immortalized human cervical (HeLa; CCL-2™) cells from the American Type Culture Collection (ATCC, Manassas, VA) used in this study were kindly provided by Dr. José das Neves from Instituto de Investigação e Inovação em Saúde (i3S). The HeLa cells were cultivated under routine culture conditions in Dulbecco’s Modified Eagle Medium (DMEM) with a high glucose concentration of 4.5 g/L. This medium was supplemented with 10% (v/v) heat inactivated Fetal Bovine Serum (FBS) and 1% (v/v) antibiotic and antimycotic from Invitrogen, MA, USA. The cells were used between passages 29 and 49 and maintained in a humidified atmosphere with 5% CO_2_ at a temperature of 37 °C.

#### Cytotoxicity assay

Cytotoxicity of the samples was assessed in the HeLa cell line according to ISO 10993–5 (2009), using PrestoBlue™ Cell Viability Reagent (Thermo Fisher Scientific, MA, USA) as per manufacturer’s instructions. The samples (MOS Parr, MOS H_3_PO_4_ and commercial D-mannose) were directly dissolved in DMEM medium and sterilized using a sterile syringe filter with a 0.22-µm pore size (Millipore, Billerica, MA, USA) at a concentration of 20 mg/mL. Decimal dilutions were performed to test the concentration range of 10.0–0.31 mg/mL. For the HeLa viability assay, suspended cells were placed into a 96-well microtiter plate at a seeding density of 1 × 10^4^ cells/well. The cells were then cultured for a period of 24 h to create a semi-confluent monolayer. Following this incubation period, the cell culture medium was removed and replaced with the samples. A medium without any samples during each incubation period served as a positive control. Conversely, a medium containing a final concentration of 10% of DMSO was utilized as a negative control. After an additional 24 h of incubation, PrestoBlue (PB) reagent was introduced to the wells and changes in cell viability were detected using fluorescence spectroscopy (Synergy H1, BioTek, California, USA). After a 2-h incubation period, the fluorescence was measured (λ excitation = 570 nm; λ emission = 610 nm).

### Adhesion assays and growth

#### Individual adhesion to vaginal epithelial cell-line

HeLa cells at a confluency of 80% were harvested and seeded at a final concentration of 2.0 × 10^4^ cells/mL in a 24-well microtiter plate, followed by immediate incubation at 37 °C with 5% CO_2_ in a humidified environment for 24 h. Subsequently, the cells were washed twice with PBS to eliminate all the antibiotic-containing mediums. *L. crispatus* and *C. albicans* which were previously cultured according to the description in section “inoculum preparation,” were centrifuged (4700 × g, 5 min, 4 °C), washed twice with PBS and resuspended in the same buffer at a multiplicity of infection (MOI) of 100. Meanwhile, the samples (MOS H_3_PO_4_, MOS Parr, and D-mannose) were diluted in DMEM medium at a non-cytotoxic concentration (2.5 mg/mL). HeLa cells were subjected to the samples and to *L. crispatus* or *C. albicans* suspension in PBS, and incubated for independent time-points: 15, 30, 60, and 120 min, under the same incubation conditions mentioned above (Rizzo et al. 2013). During each incubation period, DMEM was used as control.

#### Competition assay (simultaneous exposure) in HeLa cells

Similarly, to the above-described protocols, HeLa confluent monolayers were trypsinized and then seeded at a final concentration of 2.0 × 10^4^ cells/mL in a 24-well microtiter plate and incubated at 37 °C with 5% CO_2_ in a humidified environment. After 24 h of incubation, the medium was discarded, and the cells were thoroughly rinsed twice using PBS to eliminate any traces of antibiotic. A detailed description of the preparation of *L. crispatus* and *C. albicans* inoculums for cell inoculation can be found in section “individual adhesion to vaginal epithelial cell-line.” MOS H_3_PO_4_, MOS Parr, and D-mannose were diluted in DMEM medium without antibiotics to achieve a final concentration of 2.5 mg/mL. Subsequently, HeLa cells were exposed both samples and *L. crispatus* and *C. albicans* suspension in PBS under the same incubation conditions and time-points as previously mentioned. DMEM was utilized as a control during each incubation period.

#### Prophylaxis assay (sequential exposure) in HeLa cells

HeLa cells at a confluency of 80% were seeded according to the described in section “individual adhesion to vaginal epithelial cell-line” and incubated at 37 °C with 5% CO_2_ in a humidified environment. After a period of 24 h of incubation, the medium was subsequently disposed of, and the cells were subjected to two rounds of PBS washing to effectively eliminate any residual traces of antibiotics. Samples (MOS H_3_PO_4_, MOS Parr, and D-mannose) were diluted in antibiotic-free DMEM medium to a concentration of 2.5 mg/mL. HeLa cells were then incubated at 37 °C with 5% CO_2_ in a humidified environment with the samples and the *L. crispatus* at MOI of 100 during 2 h before infection of the vaginal cells with *C. albicans*. A comprehensive explanation regarding the preparation of *L. crispatus* and *C. albicans* inoculums for cell inoculation purposes can be found in section “individual adhesion to vaginal epithelial cell-line.” DMEM was used as control in each incubation period. After incubation, *C. albicans* was added to the cells with samples and *L. crispatus* at MOI of 100, followed by an incubation for the same time-points and incubation conditions above-mentioned.

#### Total viable count determination

At the conclusion of the cellular assays, the cell monolayers underwent two thoroughly washings with PBS to eliminate any bacteria that had not adhered to the cells. Following detachment with trypsin (TrypLE™ Thermo Fisher Scientific, Massachusetts, USA), the cells were subsequently resuspended in PBS. The same method for dilutions and plating described in section “prophylaxis assay (sequential exposure) with SVF” was used. Results were expressed as colony forming unit (CFU) per mL per HeLa cells in well, as described in Eq. (1).

1 $$[({\text{CFU}}/{\text{mL}})/(\mathrm{HeLa cells in well})] = (\frac{\frac{{\text{CFU}}}{{\text{mL}}}\mathrm{of Sample}}{\mathrm{HeLa cells in well}})$$

### Statistical analysis

The assessment of normality of the samples was conducted through implementation of Shapiro–Wilk’s test. Following this, a two-way analysis of variance (ANOVA) was executed, subsequently succeeded by Tukey’s post-test. The analyses were carried out employing the GraphPad Prism 7.04 software (Dotmatics, Boston, MA, USA). The presentation of the results was made in the form of mean values ± SD (standard deviation). Statistical significance was confirmed at a 95% confidence level. All experiments were performed in triplicate.

## Results

### Antifungal activity against *C. albicans*, *C. tropicalis*, and *C. glabrata*

**Fig. 1:**
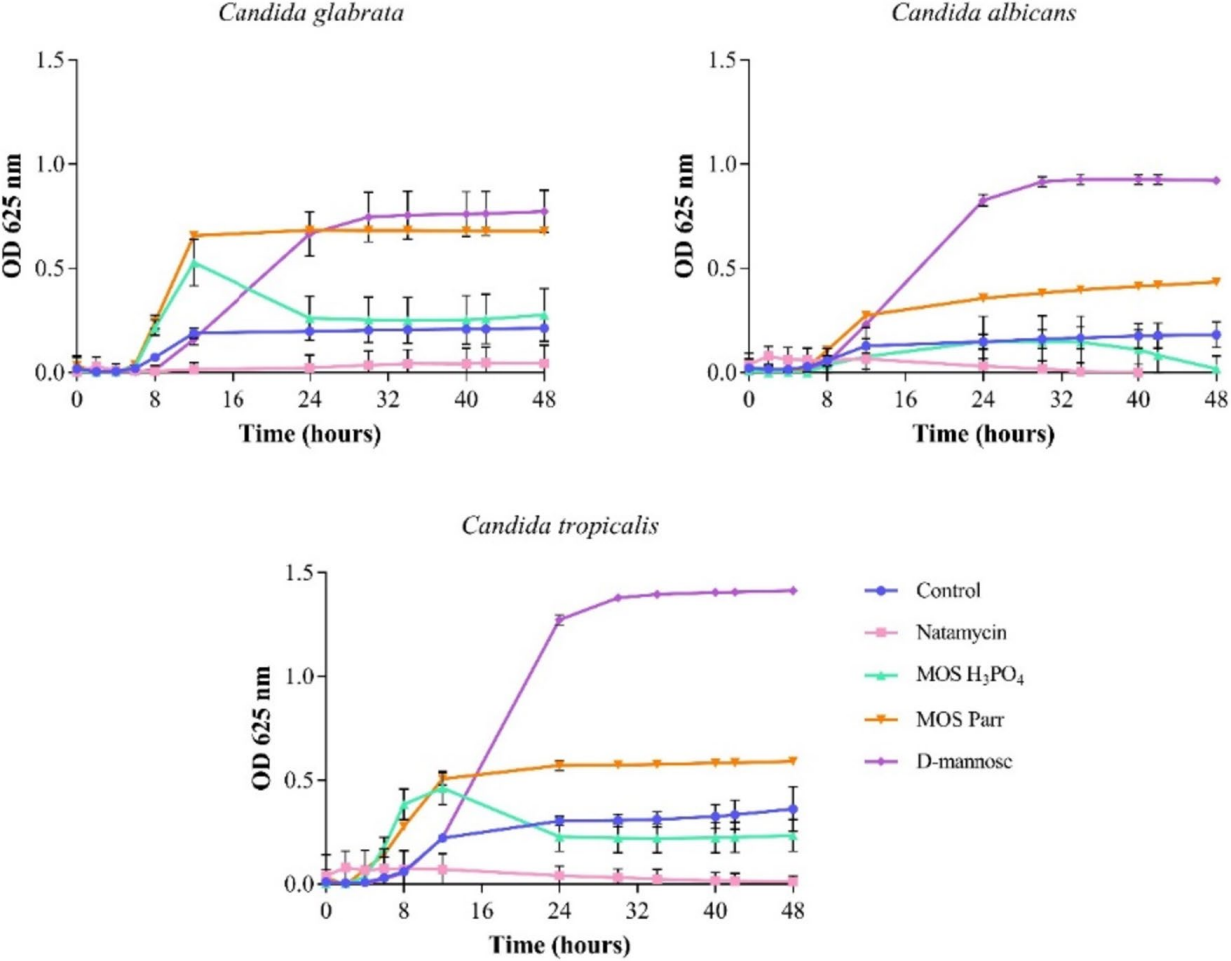
Screening of antifungal potential of several mannose-based extracts (MOS Parr, MOS H_3_PO_4_ and D-mannose, at a concentration of 2% w/v) against *C. glabrata*, *C. albicans*, and *C. tropicalis* in Muller Hinton medium

To eliminate any other contribution to further results regarding MOS probiotic effect, their potential effect upon the growth of three *Candida* sp. strains (*C. albicans*, *C. glabrata*, and *C. tropicalis*) was evaluated using a time-growth inhibition curve. As it can be seen in Fig. 1, none of the MOS extracts showed antifungal activity, when compared with the positive control (natamycin).

### Assessing microbial growth in simulated vaginal fluid: competition and prophylaxis assays

#### Screening of growth curve for each microorganism in SVF

**Fig. 2:**
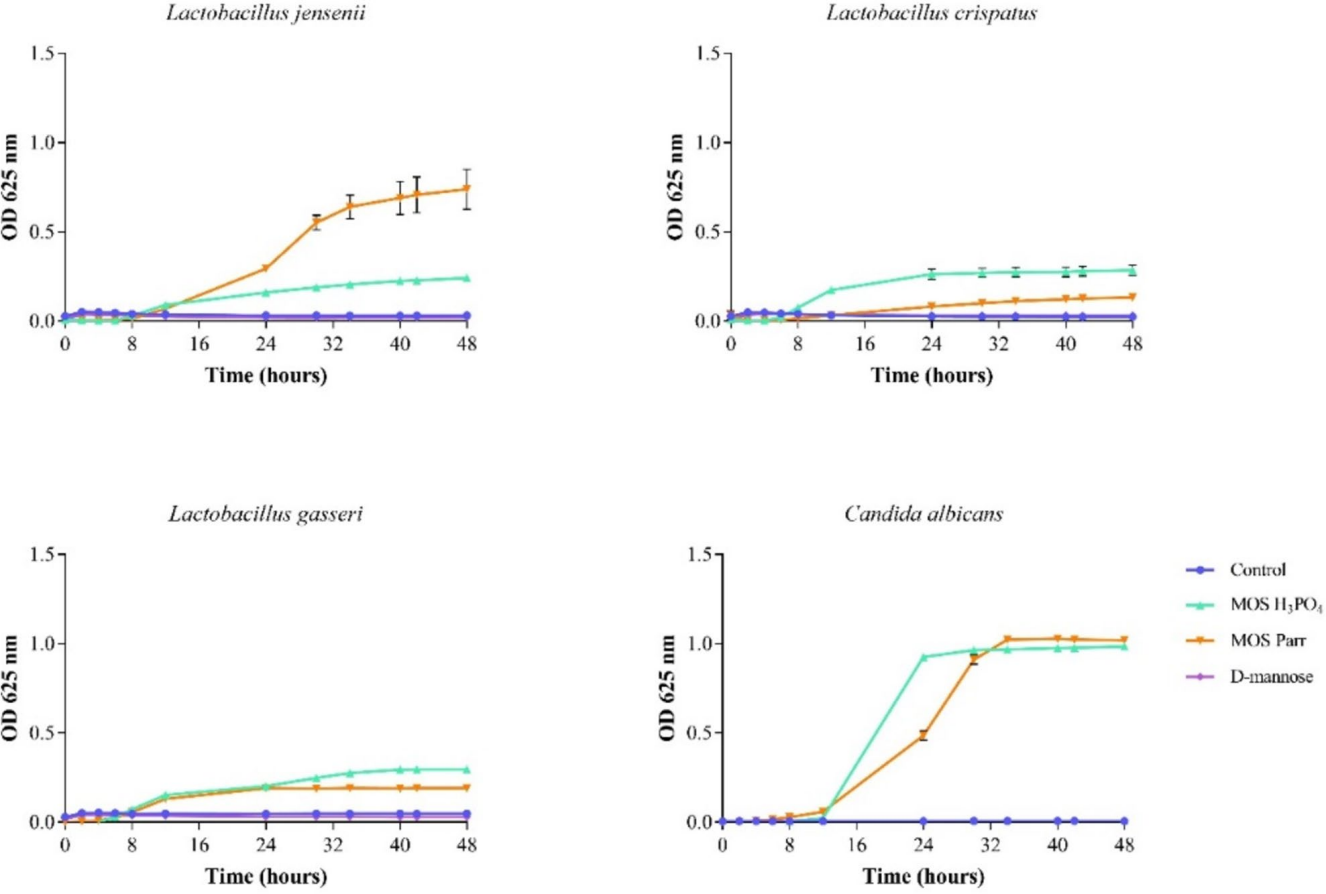
Growth curves of *L. jensenii*, *L. crispatus*, *L. gasseri*, and *C. albicans* in SVF supplemented with several mannose-based extracts (MOS Parr, MOS H_3_PO_4_, and D-mannose, at a concentration of 2% w/v):

In this work, SVF was used as growth medium, since represents a reliable mimicry of the vaginal environment. For this reason, the growth behavior of *C. albicans* and *Lactobacillus* species was previously evaluated on this fluid. The growth curves obtained in this assay can be seen in Fig. 2.

The small bacterial and fungal growth observed in the control (SVF) was already anticipated due to the poor nutrient availability of SVF as a consequence of its composition. Indeed, comparative analysis of the growth of Lactobacilli and *C. albicans* in MRS and SVF has showed that these microorganisms have a substantially smaller growth in SVF, as previously reported by Pan et al. (2020), Brandt and Barrangou (2020) and Fernandes et al. (2023). Contrary, both the growth of Lactobacilli and *C. albicans* has increased in the presence of MOS Parr and MOS H_3_PO_4_, most likely because they use can use mannose and other components of the MOS extracts.

#### Prophylaxis assay (sequential exposure) with SVF

**Fig. 3:**
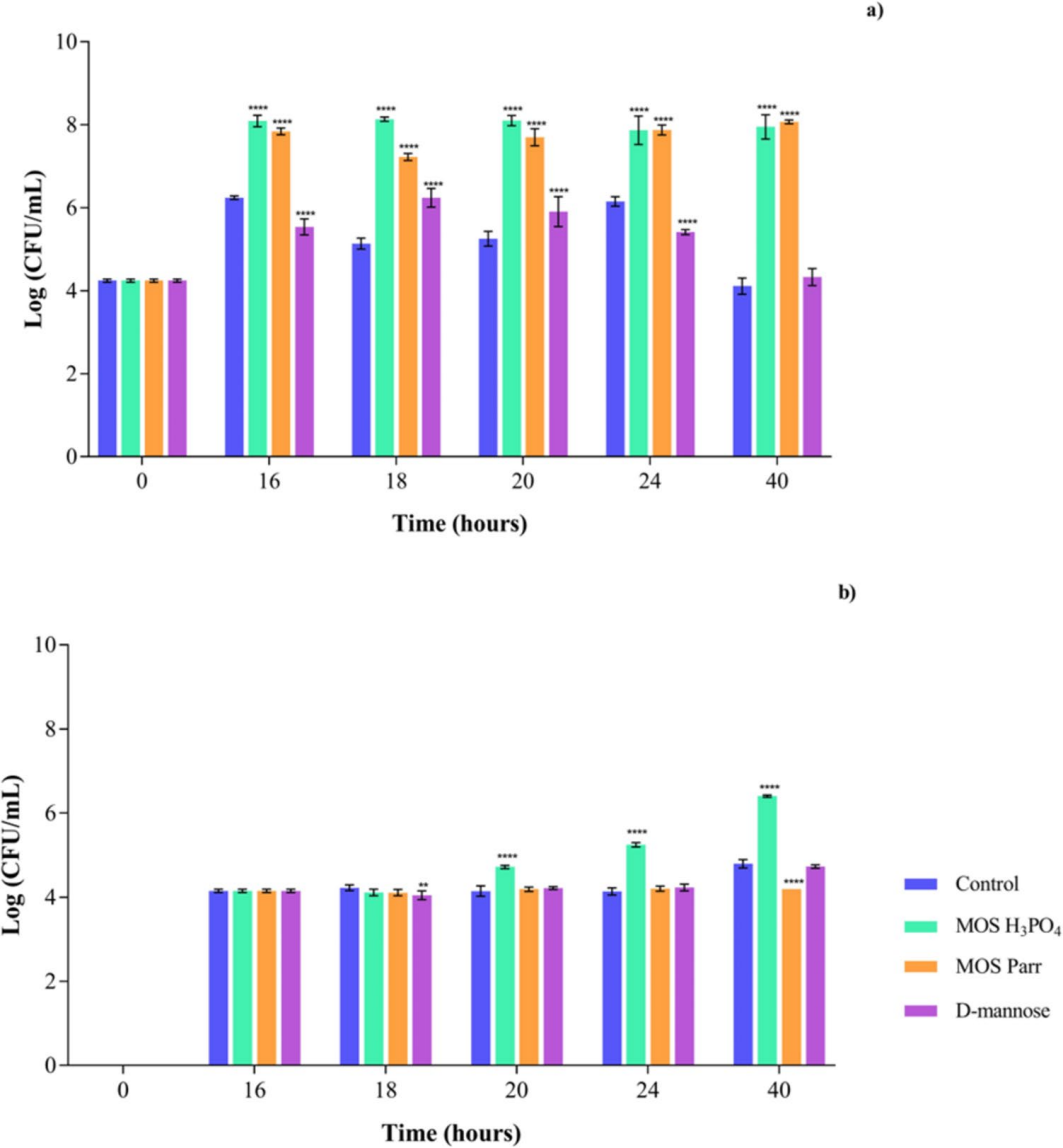
Cell viability by Log (CFU/mL) over 40 h of prophylactic effect, for **a** *Lactobacillus* sp. pool and **b** *C*. *albicans* with different extracts: D-mannose, MOS Parr, and MOS H_3_PO_4_ in SVF with 0.05% L-cysteine hydrochloride. ****p < 0.0001 and *** p < 0.001 indicate statistically significant differences between each sample and the control at each time point

After promoting the growth of the *Lactobacillus* sp. pool in SVF with the samples (MOS extracts and D-mannose) for 16 h, the *C. albicans* was added to simulate the infection, and CFU counting was performed at different time-points along the next 24 h, as previously detailed in section “prophylaxis assay (sequential exposure) with SVF.” Growth numbers of both lactobacilli and *C. albicans* along time can be seen in Fig. 3.

Figure 3a illustrates the cell viability of the *Lactobacillus* sp. pool after exposure to the different supplements (MOS extracts and D-mannose) since time zero, and exposure to the pathogen after 16 h. The cell viability remained stable throughout the 40-h assay (no significant variations in growth), displaying significant differences between the MOS extracts and the control (SVF). At 40-h incubation time, both MOS extracts exhibited a substantial increase of four log cycles in cell viability compared to the control (*p* < 0.0001*)*. Contrarily, the control and D-mannose showed a lower increase in cell viability. Figure 3b focuses on the cell viability of the selected pathogenic strain. It was observed that the cell viability remained consistent for approximately 20 h, except for the MOS H_3_PO_4_ extract. This stability in the fungal viability was somewhat predictable, since it has been reported that vaginal *Lactobacilli* can control the overgrowth of *Candida* sp. by different mechanisms such as lowering the environmental pH, secretion of antifungal compounds and inhibition of fungal adhesion to vaginal cells (Kalia et al. 2020). Due to limited reported studies with MOS isolated from *S. cerevisiae*, further research would be needed to understand the 1.1 log cycle increase at 20 h (i.e., 4 h after infection) displayed by MOS H_3_PO_4_. However, it is possible that *C. albicans* is using either mannose or other component of the MOS H_3_PO_4_ extract as a nutrient source, thus increasing its microbial growth. Differently, MOS Parr extract contributed to a smaller decrease of around 0.6 log cycles in *C. albicans* growth at the 40 h point, when compared to the control at the same time point. Differences in the impact of the two extracts are likely due to differences in their composition resulting from their different production methodologies. D-mannose exhibited similar behavior to the control (*p* > 0.05) throughout the study, maintaining cell viability at approximately between 4 and 5 log cycles.

#### Competition assay (simultaneous exposure) with SVF

**Fig. 4:**
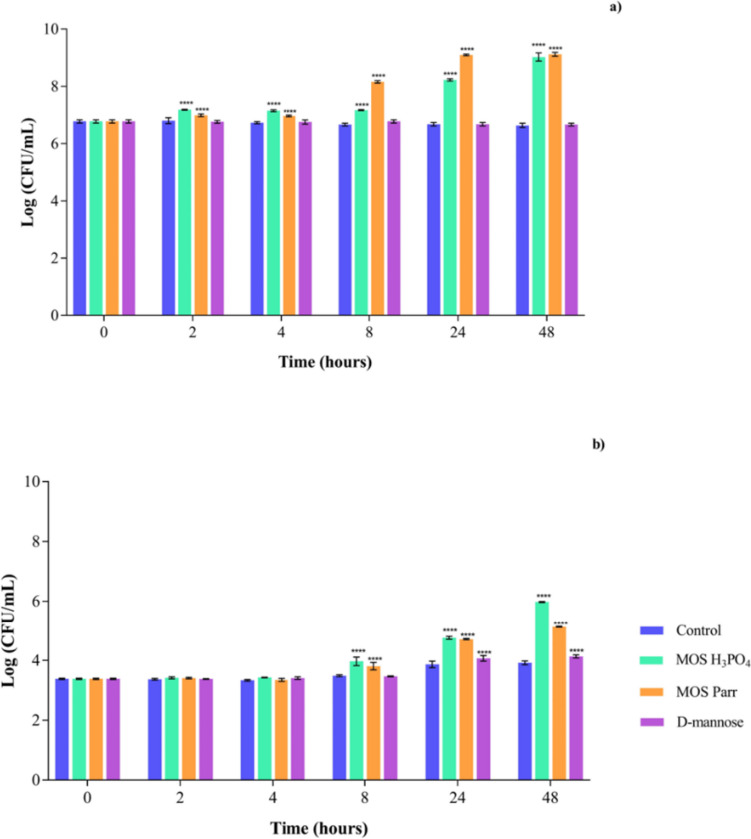
Cell viability in Log (CFU/mL) over 48 h of simultaneous exposure, for **a** *Lactobacillus* sp. pool and **b** *C*. *albicans* with different extracts: D-mannose, MOS Parr, and MOS H_3_PO_4_ in SVF with 0.05% l-cysteine hydrochloride. ****p < 0.0001 indicate statistically significant differences between the different samples and against the control at each time point

In order to simulate a typical infection scenario, and in accordance with what has previously been reported (Sobel and Chaim 1996), a concentration of 10^7^ CFU/mL for vaginal lactobacilli and 10^4^ CFU/mL for *Candida* sp. were simultaneously inoculated in SVF with the samples (MOS extracts and D-mannose). The cell viability was then monitored for a duration of 48 h, as shown in Fig. 4.

In Fig. 4a, the cell viability of vaginal lactobacilli, represented by the *Lactobacillus* sp. pool, remained stable over the 48-h period when exposed to control (SVF) and D-mannose. When considering the effect of the MOS Parr and MOS H_3_PO_4_ extracts on the vaginal lactobacilli pool, both extracts resulted in a 2.5 log cycle increase in cell viability. Regarding the impact of MOS Parr and MOS H_3_PO_4_ extracts on the fungal growth (Fig. 4b), it is perceptible that, after 48 h, both extracts also promoted *C. albicans* growth. This is likely because *C. albicans* can start using the mannose content of the MOS extracts, which is in accordance with the results obtained in the screening of growth curve for this yeast in SVF in the presence of H_3_PO_4_ and MOS Parr (Fig. 2). However, it is also possible to see that while MOS H_3_PO_4_ resulted in a 2.5 log cycle increase, only a 1.5 log cycle increase was observed for MOS Parr, further demonstrating the prebiotic potential of these extracts, specifically MOS Parr.

### Adhesion to cervical cells assays

#### Cytotoxicity

The cytotoxicity of the samples against HeLa cells was assessed by means of evaluating their influence on cell metabolism through the utilization of a cellular viability dye. As per the ISO 10993–5 (2009) standard, a sample can be identified as cytotoxic when inducing a metabolic inhibition percentage above 30% is detected. MOS Parr (23.30 ± 3.40%) and MOS H_3_PO_4_ (− 11.69 ± 6.04%) exhibited no cytotoxicity at the concentration of 2.5 mg/mL, with no metabolic inhibitions being observed. Commercial D-mannose exhibited no cytotoxicity up to 2.5 mg/mL (− 9.51 ± 1.85%). In fact, the negative values illustrate an increase in cell metabolism observed in the presence of MOS H_3_PO_4_ and D-mannose. Differences observed in the cellular viability of HeLa cells when exposed to MOS Parr and MOS H_3_PO_4_ may have origin in the structure/composition of the extracts.

#### Adhesion to vaginal epithelial cells

**Fig. 5:**
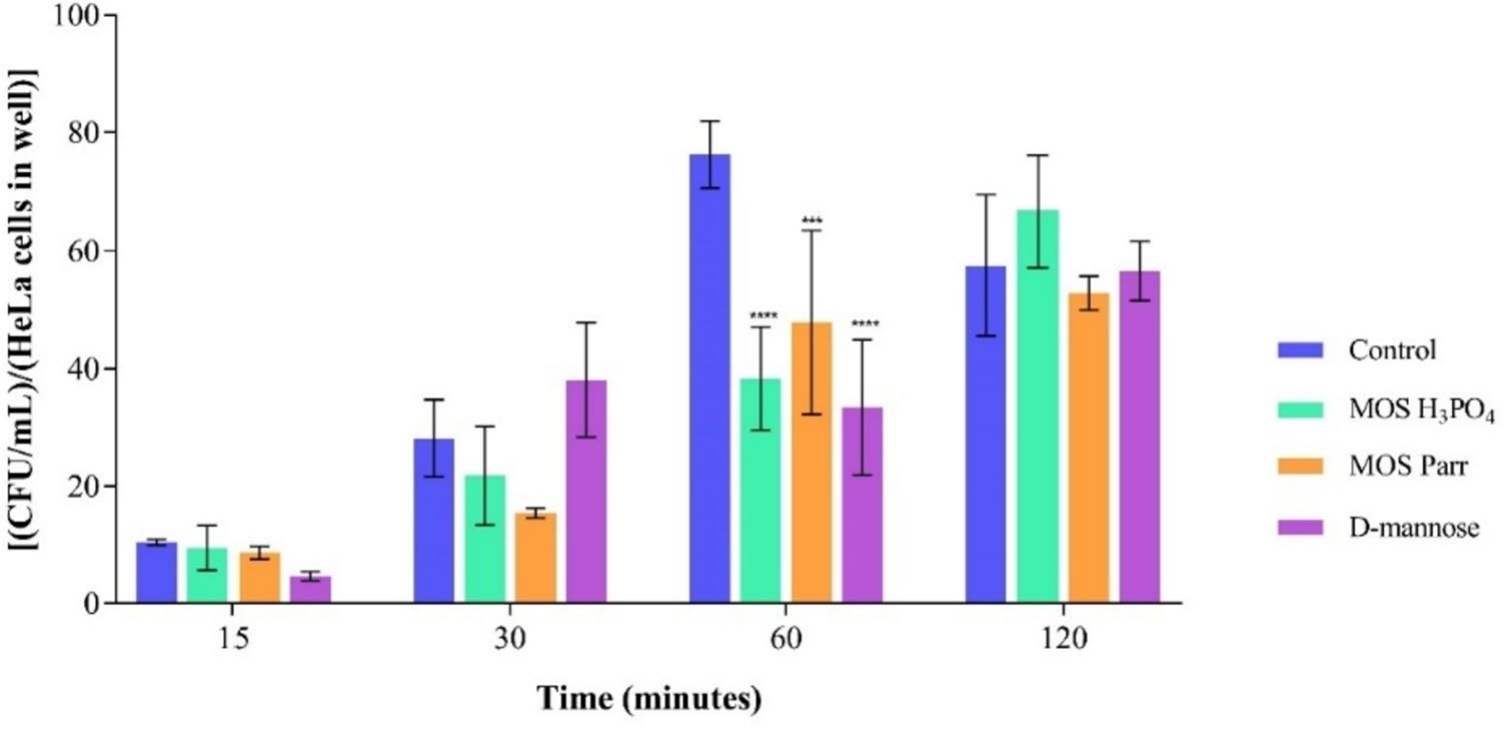
CFU/mL/ HeLa cells in well over 120 min of individual adhesion, for *C. albicans* with different extracts: D-mannose, MOS Parr, and MOS H_3_PO_4_. **** *p* < 0.0001 and *** p < 0.001 indicate statistically significant differences between the different samples and against the control at each time point

In order to increase the degree of complexity and, thus, obtain a more realistic approach of the biological human complexity, the extracts were also assessed for their ability to induce or prevent the adherence of *C. albicans* to HeLa cells. This assay was performed according to the description in section “individual adhesion to vaginal epithelial cell-line,” and pathogen cell numbers were normalized by the number of HeLa cells in each well. Results are depicted in Fig. 5.

Figure 5 illustrates the initial adaptation of *C. albicans* to the cellular system, and the fast-growing rate by which adhesion is observed. In the control group, *C. albicans* reached its peak at 60 min and declined by 120 min. In contrast, all other samples only peaked at 120 min. Despite these results, there were no statistically significant differences (*p* > 0.05) at 120 min, suggesting that our samples cannot delay the adhesion of *C. albicans* within the studied time. According to Mayer et al. (2013), *C. albicans*’ capacity to infect and adhere to various host niches is supported by a diversified set of virulence and fitness variables. The manifestation of virulence in a pathogenic organism is attributed to a multitude of factors, including the morphological transformation from yeast to hyphal forms, the synthesis of adhesive and invasive molecules on the cell exterior, and the ability to respond to mechanical stimuli, known as thigmotropism (the capacity to detect and respond to surface contour changes), biofilm development, phenotypic switching, and the release of hydrolytic enzymes (Davies et al. 1999). Among a multitude of fitness characteristics, *C. albicans*’ potent food acquisition mechanisms, metabolic flexibility, prompt adaptability to changes in ambient pH, robust stress response machinery and form biofilms on abiotic or biotic surfaces are major virulence factor (Tester et al. 2012).

As observed during the assays in SVF, the viability of both *Lactobacillus* and *Candida* species depended not only on the extract to which they were exposed, but also on potential interactions between microorganisms (e.g., growth of *Candida* in SVF with MOS extracts was similar for the two extracts when the yeast was cultivated alone, but different when lactobacilli were also present). Therefore, the subsequent step of the study focused on testing the ability of MOS extracts to inhibit the adhesion of *C. albicans* to HeLa cells in the presence of *L. crispatus*, again in a prophylaxis or competition mode assay.

#### Competition (simultaneous exposure) in HeLa cells

**Fig. 6:**
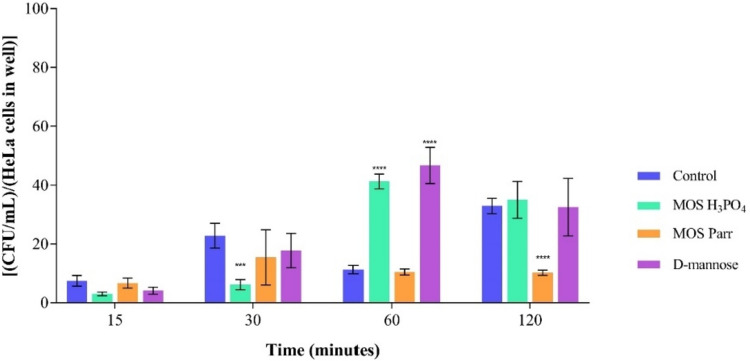
CFU/mL/ HeLa cells in well over 120 min of simultaneous exposure, for *C. albicans* with different extracts: D-mannose, MOS Parr, and MOS H_3_PO_4_. ****p < 0.0001 and *** p < 0.001 indicate statistically significant differences between the different samples and against the control at each time point

This study evaluates if MOS promotes the inhibition of *C. albicans* adhesion on HeLa cells in synergy with *L. crispatus*. This species was selected as the most representative from the vaginal lactobacilli since it would be very complicated to work with the previous pool of *Lactobacillus* sp. in the cell line. Figure 6 demonstrates the inhibitory effect of the extracts in synergy with *L. crispatus* on the adhesion of *C. albicans* to HeLa cells.

With respect to MOS Parr, results show a significant reduction in *C. albicans* adhesion when inoculated simultaneously with vaginal lactobacilli, with an adhesion result of 10.21 ± 0.88 CFU/mL/HeLa cells in the well. This indicates that the combination of MOS Parr extract and *L. crispatus* has an inhibitory effect on the adhesion of *C. albicans* to the HeLa cells. The control group, which consists of *L. crispatus* alone, demonstrated an adhesion result of 32.92 ± 2.60 CFU/mL/HeLa cells in the well. Results observed for MOS Parr are in line with those previously obtained in the microbiological prophylaxis assay, showing that MOS Parr has a negative impact on *Candida* growth and adhesion on HeLa vaginal cells, while MOS H_3_PO_4_ has no effect on the virulence inhibition of *Candida*.

#### Prophylaxis assay in HeLa cells

**Fig. 7:**
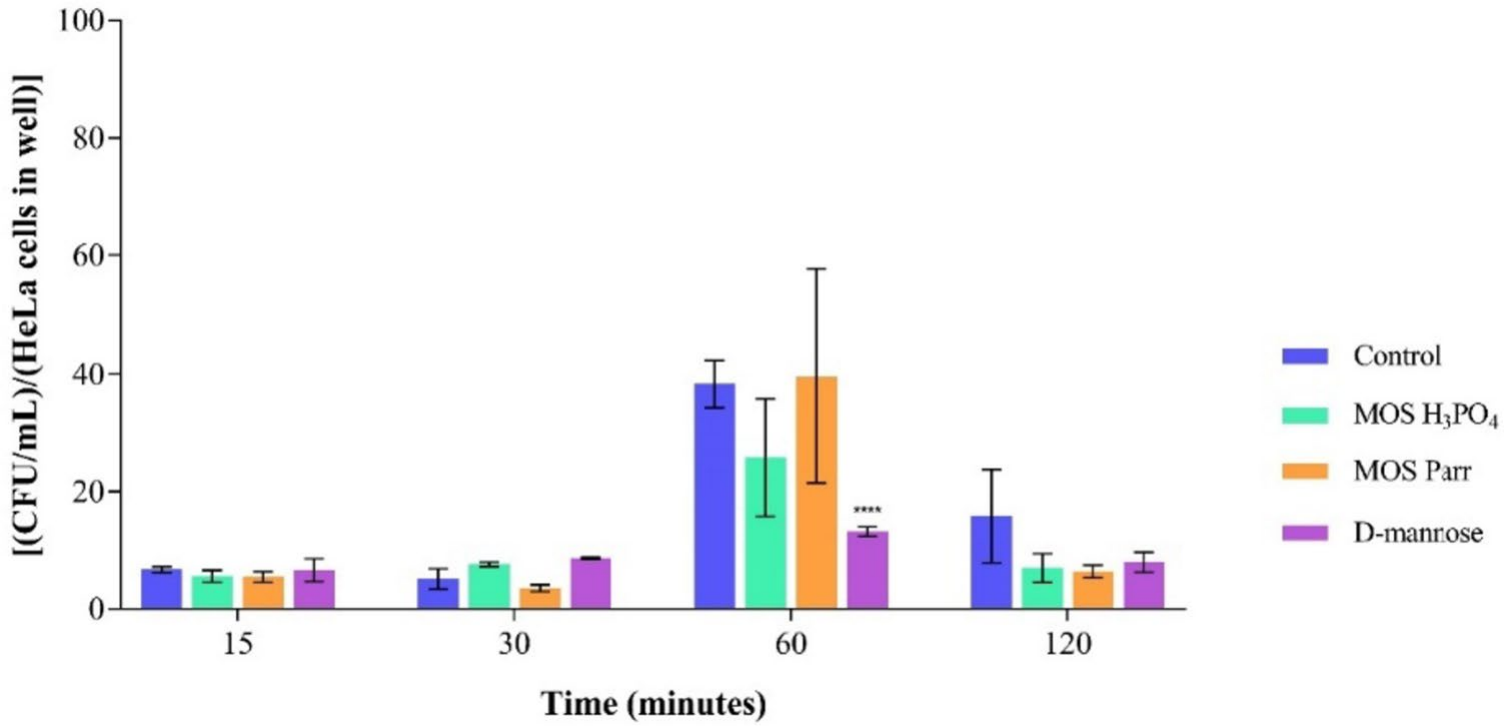
CFU/mL/ HeLa cells in well over 120 min of prophylaxis assay, for *C. albicans* with different extracts: D-mannose, MOS Parr, and MOS H_3_PO_4_. **** *p* < 0.0001 indicate statistically significant differences between the different samples and against the control at each time point

According to the literature, *Lactobacillus* sp. species present in the female urogenital system serve as a formidable barrier to infections and play a significant role in controlling the vaginal microbiota by engaging in competition with other microorganisms for epithelial cell adherence and displacing pathogenic biofilm (Boris et al. 1998; Saunders et al. 2007), and/or inhibiting the growth of potential pathogens (Strus et al. 2005; Atassi et al. 2006; Spurbeck and Arvidson 2008). Furthermore, supernatants from *L. gasseri* and *L. crispatus* reduce the *C. albicans* ability to adhere to HeLa cells (Matsuda et al. 2018). Thus, the prophylaxis assay aims to simulate the probiotic/prophylactic effect of vaginal lactobacilli (*L. crispatus*) in synergy with MOS extracts against the adhesion of *C. albicans*. The results are represented in Fig. 7.

In this assay, a robust population of lactobacilli was established in the cells for 120 min, after which the pathogen was inoculated (5.08 × 10^5^ CFU/ml). At the end of 120 min, the production of compounds such as biosurfactants (De Gregorio et al. 2020), which prevent the adhesion by *L. crispatus* may occur, which, in synergy with MOS extracts affects the adhesion of *C. albicans* and the subsequent proliferation and infection of the cells by this pathogen. Based on the analysis of Fig. 7, it can be inferred that the presence of an established population of vaginal lactobacilli has a detrimental effect on the adhesion of *C. albicans*, particularly when in synergy with MOS extracts (despite no statistically significant differences were observed at 60 min and 120 min between the MOS extracts and the control). The adaptation of *C. albicans* can be observed between the initial 15 and 30 min, as it adjusts to the presence of the lactobacilli population. However, at the end of 120 min, the adhesion is inhibited, and this inhibition is positively affected by the MOS extracts and D-mannose (MOS H_3_PO_4_ = 7.00 ± 2.43, MOS Parr = 6.42 ± 1.06, D-mannose = 7.96 ± 1.69 CFU/mL/HeLa cells in the well) in synergy with *L. crispatus* established population, mainly, even though there is no statistically significant evidence between the tested samples and the control (15.83 ± 7.94 CFU/mL/HeLa cells in the well).

## Discussion

The present study aimed to assess the potential of yeast mannan oligosaccharides (MOS) as a synergetic therapy with probiotics to promote *Lactobacillus* sp. (*L. crispatus*, *L. gasseri*, and *L. jensenii*) population in vaginal flora and simultaneously regulate the uncontrolled growth of *C*. *albicans*, thus promoting a healthy vaginal microbiome. Putative antifungal activity of MOS extracts was evaluated against *Candida* species strains (*C. glabrata*, *C. albicans*, and *C. tropicalis*).

Commercial D-mannose was used for comparison with MOS, as these are composed essentially of mannose monomers. *Candida* strains exhibited the highest growth rate when exposed to mannose, especially *C. tropicalis* and *C. albicans*. MOS Parr was the second-best growth promoting sample, able to promote growth of all strains of *Candida* sp. On contrary, MOS H_3_PO_4_ extract did not substantially promote the growth of the *Candida* strains, as evidenced by the similar OD values read when compared to the medium control, which indicates prevalence of the yeast but not the promotion of its growth. Thus, it is possible to infer that, neither mannose nor MOS extracts present antifungal activity against the above tested *Candida* strains. Despite literature studies on the antifungal activity of MOS is scarce, other oligosaccharides such as chitosan and alginate oligosaccharides have been studied and demonstrated antifungal activity against *Candida* sp. (Ganan et al. 2019; Powell et al. 2023).

A healthy vaginal environment is frequently associated with microbiota dominated by *L. crispatus*, *L. gasseri*, and/or *L. jensenii* (Burton et al. 2003; Petrova et al. 2017). Nevertheless, some woman present a microbiome with more facultative and anaerobe bacteria (Ravel et al. 2011), such as *Prevotella* or *Gardnerella* (Hickey et al. 2012; Green et al. 2015), which are also considered as healthy, typical vaginal flora in asymptomatic women, and their presence does not always indicate illness (Zangl et al. 2020). However, for the present study, the three above-mentioned lactobacilli were considered as the typical microbiota from healthy vaginal environment. Concerning *Candida* species, *C. albicans* was selected as the most representative pathogenic yeast (Turner and Butler 2014).

Since SVF was used in this study as a good proxy for the vaginal environment (Owen and Katz 1999; Borges et al. 2012, 2013), the behavior of the microorganisms under study had to be, initially, evaluated on this fluid, i.e., growth curves needed to be performed in SVF to define infection times during prophylaxis assays; results are depicted in Fig. 2. When comparing the growth of the selected microorganisms in SVF with their growth in MH medium, most microorganisms present a lower growth, particularly in the control, where species only have access to SVF components. This is not surprising since SVF is not rich in nutrients, namely sugars. By analyzing the maintenance curves in SVF of *L. gasseri* and *L. crispatus*, it can be observed that both vaginal lactobacilli maintain its viability in SVF supplemented with MOS extracts and D-mannose. Regarding *L. jensenii* and *C. albicans*, the two MOS extracts showed a substantial promotion of bacterial growth, while in D-mannose and in the control, only the maintenance of the tested microorganisms was observed. Upon the conclusion of this study, valuable insights were gathered, confirming the viability of all microorganisms for a duration of 48 h in SVF. This fluid served as the designated vaginal environment to carry out the presented assays, namely competition and prophylaxis, within the simulated vaginal setting.

Moreover, this research was carried out to complement the results presented in Supplementary Data Figure S1, pertaining to the growth curves of vaginal lactobacilli in MRS broth medium, which is known to be an optimal growth medium for these microorganisms. By analyzing these growth curves, we were able to identify the exponential phase (approx. 16 h), which corresponds to a vaginal lactobacilli density similar to that found in the vaginal flora (approximately a density of 10^7^ CFU/mL, Sobel and Chaim 1996). This information played a crucial role in the subsequent experiments, as it allowed us to prepare the pre-infection vaginal environment in a consistent and relevant manner. By identifying the start of the exponential phase and understanding the growth curves of lactobacilli in MRS broth medium, we were able to establish the appropriate conditions to mimic the vaginal flora and to ensure the validity of the studies presented.

The protective effect of vaginal *Lactobacillus* strains is typically evaluated in vitro through its capacity to attach to the vaginal epithelium, as well as its antibacterial efficacy against *Gardnerella vaginalis* and *C. albicans*, probably through the production of hydrogen peroxide (H_2_O_2_) and weak organic acids as well (Klebanoff et al. 1991; McGroarty et al. 1992). Single strains of vaginal lactobacilli inhibit *Candida* sp. development in vitro (Strus et al. 2005), and this has also been observed in vivo (Drutz 1992). However, the mechanism underlying this growth suppression remains unknown. Thus, following the study of the individual behavior of each microorganism in SVF supplemented with MOS extracts, a prophylaxis study was designed to simulate the potential preventive effect of MOS against *C. albicans* infection in a “healthy” vaginal environment, i.e., already containing a population of different *Lactobacillus* species. This study allowed for the examination of cell viability in the *Lactobacillus* sp. pool, indicating a prebiotic effect of MOS, as well as the viability of the pathogenic yeast over a 40-h period (Fig. 3). For this purpose, the *Lactobacillus* sp. pool was initially placed in SVF with 2% (w/v) of each extract for 16 h. The 16-h point was chosen to start mimicking the infection by *C. albicans* (Supplementary Data Figure S1), because in this set time the vaginal lactobacilli reach the desired concentration to resemble the vaginal microbiota, approximately 10^7^–10^9^ CFU/mL (Sobel and Chaim 1996). Subsequently, 2% (v/v) of *C. albicans* was added to simulate the infection, and the study continued up to 40 h, with several sampling points.

The *Lactobacillus* sp. pool growth was promoted in the presence of both MOS extracts, reaching a cell viability of about eight log cycles from 16 to 40 h of incubation. This shows the potential prebiotic effect of the MOS extracts, as they have clearly induced the growth of the vaginal lactobacilli pool, with no statistically significant distinction between the two types of extracts. On the other hand, the two MOS extracts have induced a different outcome in *C. albicans* viability: whereas MOS H_3_PO_4_ promoted the growth of pathogenic strain, MOS Parr did not kill *C. albicans* but prevented an increase in its growth, thus maintaining a cell viability of around four to five log cycles until the end of the study (40 h), which was the same compared to the control (Fig. 3b). However, in the absence of lactobacilli (Fig. 2), MOS extracts have no differentiated effect on *C. albicans* growth, and thus we hypothesize that the changes observed in Fig. 3b must be a result of the interference of MOS extracts in the relationship between vaginal lactobacilli and *Candida* sp.

One theory is that, since it is known that vaginal lactobacilli may negatively impact *C. albicans* growth, as discussed above, it is possible that MOS H_3_PO_4_ protects *C. albicans* from these mechanisms in some way not currently understood. In addition, it remains unclear how the fungus might utilize the MOS and if it could alter fungal metabolism. To the best of our knowledge, this is the first time this behavior is observed, and further studies are needed to understand the underlaying mechanisms associated with it.

To simulate VVC infections induced by *C. albicans*, both the *Lactobacillus* pool and *C. albicans* strains were co-inoculated at the specified concentrations in SVF enriched with the various extracts (Fig. 4). Ideally, the desired outcome of this assay would be the reduction or, at least, maintenance (no increase) of *C. albicans* numbers, while the growth of the *Lactobacillus* sp. pool would be promoted. In fact, the coexistence of *Lactobacillus* strains and *Candida* strains in the vaginal epithelium of healthy women has been reported (Falagas et al. 2006). In vitro and clinical trials have shown positive results regarding the efficacy of specific *Lactobacillus* strains against *C. albicans.* Nevertheless, it is important to note that different probiotic strains can have distinct qualities and effects on *C. albicans*, and thus, findings from research evaluating one *Lactobacillus* strain should not be generalized to others. However, although *Candida* growth has been observed in both MOS extracts, *Candida* grown in the presence of MOS Parr still has shown less viability than that grown with MOS H_3_PO_4_, supporting our previous remarks about the protective effects that MOS H_3_PO_4_ may have.

After ensuring that MOS extracts exerted non-cytotoxicity effect on HeLa cells, they were evaluated on their capacity to promote or inhibit the adhesion of *C. albicans* to cervical cells (Fig. 5). HeLa cells are human female cervical cancer cells (Rizzo et al. 2013) widely used as a model system of the vaginal environment (Cautela et al. 2019; Facchinatto et al. 2021) and to investigate the impact of vaginal lactobacilli on *C. albicans* adhesion capacity (Rizzo et al. 2013; Calonghi et al. 2017). The synergetic effect of MOS extracts with *L. crispatus* on the adhesion inhibition of *C. albicans* to the cervical cells was evaluated resorting, once more, to competition (Fig. 6) and prophylaxis (Fig. 7) assays. To minimize the variations in the experimental conditions, assays were performed in the same day, with the same samples and the same inoculum concentration of *L. crispatus*, *C. albicans* and HeLa cells (and thus the same MOI).

According to Niu et al. (2017), *L. crispatus* is one of the most predominant microorganisms on the vaginal microbiota and has been shown to decrease the virulence of *C. albicans* and increase the local immune response of the vaginal epithelium by modulating the immune cytokine and chemokine profile. Another study by Sun et al. (2023) has also shown that vaginal lactobacilli can inhibit the adhesion of *C. albicans* to vaginal epithelium.

Lactobacilli adhesion to the epithelium marks the initial step in forming a barrier against undesirable microbial colonization (Borchert et al. 2008). Figure 7 illustrates the synergistic effect of MOS extracts and *L. crispatus*, evident when comparing with Fig. 5, where *C. albicans*, in isolation, shows no inhibition of adhesion in the presence of extracts. This synergy leads to decreased *C. albicans* adhesion after 120 min, promoting inhibitory effects. Probiotic microorganisms are recognized as an alternative therapy for *Candida* infections, extensively studied in urogenital, gastrointestinal, and oral infections (Meurman 2005; Matsubara et al. 2012, 2016; Hu et al. 2013; Roy et al. 2014; Li et al. 2014; Kovachev and Vatcheva-Dobrevska 2015; Ishikawa et al. 2015; Kraft-Bodi et al. 2015). Lactobacilli produce antibacterial metabolites, including bacteriocins, cyclic dipeptides, enzymes, fatty acids, biosurfactants, and organic molecules like reuterin 3-Phenyllactatic acid and acetyl-beta-carboline (MacAlpine et al. 2021). These metabolites alter fungal physiology, inducing oxidative stress, depleting ATP, causing cytotoxicity and suppressing growth. Some weaken the fungal cell structure, inducing changes in shape, membrane permeability, and death, while biosurfactants prevent adhesion to mucosal surfaces (Vazquez-Munoz and Dongari-Bagtzoglou 2021). *Lactobacilli* may produce inorganic chemicals such as hydrogen peroxide, exhibiting antibacterial action against a wide range of bacteria and fungi (Strus et al. 2005; Crowley et al. 2013; Siedler et al. 2019; Ribeiro et al. 2020; Lipinska-Zubrycka et al. 2020). *L. gasseri* and *L. crispatus* strains are reported to prevent *C. albicans* adhesion through alterations in polar lipid structure, physical characteristics, and α5β1 integrin exposure (Calonghi et al. 2017), thus creating a crucial barrier against microbial colonization (Ocaña and Nader-Macías 2001).

There is limited information available regarding the specific use of MOS in inhibiting the adhesion of *C. albicans* on vaginal cells. Nevertheless, according to Al-Ghazzewi et al. (2007), hydrolysed glucomannan increases the growth, metabolism, and antibacterial capabilities of probiotic microorganisms, including vaginal healthy lactobacilli strains (Sutherland et al. 2009), implying that hydrolysed glucomannan might be used for vaginal treatment. A study conducted by Tester et al. (2012) showed hydrolysed glucomannan’s symbiotic potential to restore the healthy microbiota of vagina treated with antifungal drugs. Tester and colleagues also found that inserting pessary capsules containing hydrolysed glucomannan inserted into the vagina aided in the recovery and optimization of healthy vaginal microbiology, hence preventing future infection. Therefore, a possible reason for the obtained results in Fig. 6 is that MOS H_3_PO_4_ either protects *Candida* from these antifungal mechanisms, or *L. crispatus* is not as promoted by MOS H_3_PO_4_ as it is by MOS Parr, resulting in the observed patterns. However, further studies are needed to address this issue and draw definitive conclusions.

In summary, the present study has shown that SVF was an adequate medium to mimic the vaginal fluid to grow/maintain *Lactobacillus* sp. pool and *Candida* strains. Both MOS extracts appear to have a positive prebiotic effect in *Lactobacillu*s strains in SVF medium. Additionally, *C. albicans* growth is negatively impacted in the presence of MOS Parr, while unaffected in the presence of MOS H_3_PO_4_, which raises the possibility that MOS H_3_PO_4_ may exert some sort of protective “umbrella” over *Candida* sp. and protect it from lactobacilli antifungal mechanisms. Furthermore, in cell-line assays, MOS Parr extract in synergy with *L. crispatus* has also demonstrated an inhibitory effect on the adhesion of the *C. albicans*. This inhibition is likely due to a potentiation effect of lactobacilli antifungal properties by MOS Parr, since this situation does not occur when adhesion of *C. albicans* occurs in HeLa cells alone, where none of the MOS extracts promote *C. albicans* adhesion inhibition. More research is, however, still needed to fully understand the mechanisms by which MOS may affect the adhesion of *C. albicans* to host cells and whether it has potential as a therapeutic agent for preventing vaginal infections. Additionally, besides *L. crispatus*, it would be interesting to see if a similar trend is observed with another *Lactobacillus* sp., such as *L. gasseri* and *L. jensenii*, and their impact in the adhesion of *C. glabrata* and *C. tropicalis* in the presence of the MOS extracts.

## Supplementary Information

Below is the link to the electronic supplementary material.

Supplementary file1 (PDF 122 KB)

## Data Availability

The datasets generated and/or analyzed during the current study are available from the corresponding author on reasonable request.
