# Supplementary material for: Vaginal prevention of Candida albicans: synergistic effect of lactobacilli and mannan oligosaccharides (MOS)
Source: Appl Microbiol Biotechnol. 2024 Jan 9;108:73. doi: 10.1007/s00253-023-12909-2 (PMC10776728; doi:10.1007/s00253-023-12909-2)
Supplement: Supplementary file 1 [file 253_2023_12909_MOESM1_ESM.pdf]

Vaginal prevention of *Candida albicans*: synergistic effect of lactobacilli and mannan oligosaccharides (MOS)

Margarida Faustino [1], Joana Odila Pereira [1,2*], Ana Margarida Pereira [1,2], Ana Sofia Oliveira [1], Carlos M. H. Ferreira [1,2], Carla F. Pereira [1], Joana Durão [1,2], Manuela E. Pintado [1], Ana P. Carvalho [1*]

[1] Universidade Católica Portuguesa, CBQF–Centro de Biotecnologia e Química Fina–Laboratório Associado, Escola Superior de Biotecnologia, Rua Diogo Botelho 1327, 4169-005 Porto, Portugal.

[2] Amyris Bio Products Portugal Unipessoal Lda, Portugal

Electronic Supporting Information

All correspondence should be addressed:

Ana P. Carvalho: apcarvalho@ucp.pt & Joana Odila Pereira: jodila@ucp.pt

Supplementary material

Supplementary material S1 – List of bacteria and yeast strains

Table S1. Strains used in this study.

Microorganism		Catalogue #	URL
**Bacteria**	*Lactobacillus jensenii*	20557	https://www.dsmz.de/collection/catalogue/details/culture/DSM-20557
	Lactobacillus crispatus	20356	https://www.dsmz.de/collection/catalogue/details/culture/DSM-20356
	Lactobacillus gasseri	20243	https://www.dsmz.de/collection/catalogue/details/culture/DSM-20243
**Yeast**	*Candida albicans*	3454	https://www.dsmz.de/collection/catalogue/details/culture/DSM-3454
	Candida glabrata	28718	https://www.dsmz.de/collection/catalogue/details/culture/DSM-28718
	Candida tropicalis	11952	https://www.dsmz.de/collection/catalogue/details/culture/DSM-11952

Supplementary material S2 - Reagents/Chemicals Used in Simulated Vaginal Fluid

Table S2. Simulated Vaginal Fluid at 4.2 pH.

Reagent	Concentration g/L	Purchased from
**Sodium Chloride**	3.5	Honeywell Fluka, Seelze, Germany
**Potassium Hydroxide**	1.4	Merck, Darmstadt, Germany
**Calcium Hydroxide**	0.2	Sigma, St. Louis, MO, USA
**Bovine serum albumin**	0.02	Sigma, St. Louis, MO, USA
**Lactic Acid**	2.0	Sigma, St. Louis, MO, USA
**Acetic Acid**	1.0	Sigma, St. Louis, MO, USA
**Glycerol**	0.2	Fisher Scientific, Loughborough, UK
**Urea**	0.4	Sigma, St. Louis, MO, USA
**D- Glucose**	5.0	Merck, Darmstadt, Germany
**L-cysteine hydrochloride**	0.05	Sigma, St. Louis, MO, USA

Supplementary material S3 – Growth curves in MRS broth medium.

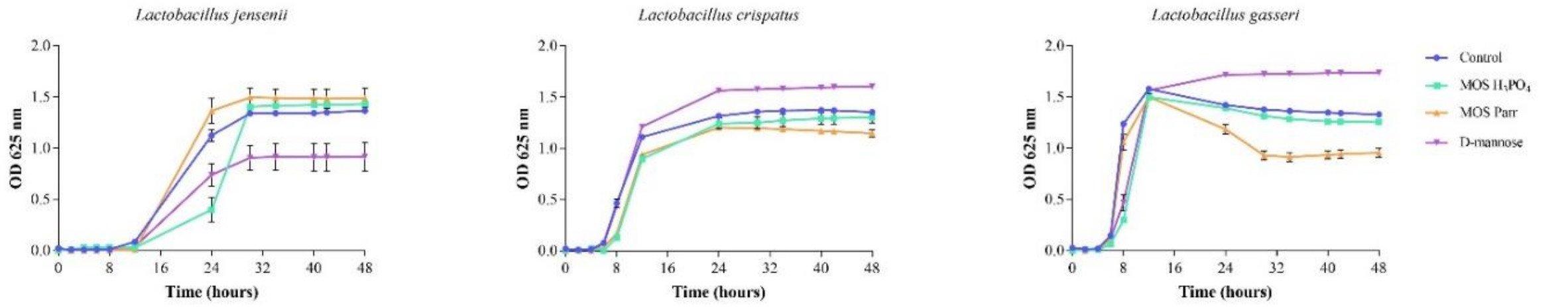

Figure S1. Growth curves of *Lactobacillus jensenii*, *L. crispatus* and *L. gasseri* and in MRS broth supplemented with several mannose-based extracts.
